# Unveiling the Humanizing and Therapeutic Values of Live Music in Healthcare Settings: A Scoping Review

**DOI:** 10.3390/healthcare14121805

**Published:** 2026-06-22

**Authors:** Conrado Carrascosa-Lopez, Miriam Serrano-Soliva, María De-Miguel-Molina, Blanca De-Miguel-Molina, Daniel Catala-Perez

**Affiliations:** 1 Management Department, Universitat Politècnica València, Camino Vera s/n, 46022 Valencia, Spain; mademi@omp.upv.es (M.D.-M.-M.); bdemigu@omp.upv.es (B.D.-M.-M.); dacapre@ade.upv.es (D.C.-P.); 2 Professional Conservatory of Music Buñol, 46360 Valencia, Spain; mserrano@bunyol.es

**Keywords:** live music, healthcare, hospital, therapeutic values, scoping review

## Abstract

Background: Live music, understood as real-time musical performance delivered in the physical presence of patients or other participants, is increasingly incorporated into healthcare settings as an arts-based, non-pharmacological practice intended to support well-being and humanize care. While previous reviews have examined a broad range of music-based interventions in healthcare, limited attention has been given specifically to live music, its contextual characteristics, and the values attributed to its use within hospital environments. Objectives: This scoping review aims to map and synthesize the literature on live music in healthcare settings, focusing on clinical contexts, populations involved, and the therapeutic, psychosocial, and environmental values reported. Methods: A scoping review was conducted following the framework of Arksey and O’Malley. Searches were performed in Web of Science, Scopus and Pubmed using terms related to live music and healthcare settings. Studies published in English or Spanish over the past 20 years were considered. After screening titles, abstracts, and full texts, 81 studies met the inclusion criteria. Results: The studies covered diverse hospital units and patient groups, particularly oncology, neonatal and intensive care, palliative care, and haemodialysis. Reported outcomes were mainly psychological and emotional, including reductions in anxiety, stress, and distress, alongside improvements in mood, well-being, and quality of life. Cognitive, physiological, and environmental benefits were also identified, emphasizing the role of live music in creating supportive and humanized care environments. Most studies were conducted in Europe and North America. Conclusions: Live music is widely implemented in healthcare settings and is associated with benefits extending beyond symptom reduction to experiential and humanizing dimensions of care. This scoping review provides an overview of the existing evidence base and identifies directions for future research in arts and health.

## 1. Introduction

There has been growing interest in non-pharmacological interventions as adjuncts to medical care. Among these, music therapy and acupuncture are widely used. Since the early 21st century, there has been a significant increase in research on the effects of the arts on health and well-being [1]. Similarly, recent bibliometric studies, such as [2], reveal that publications on music therapy have experienced substantial growth in recent years.

Music therapy is applied across diverse demographic profiles and fields, ranging from neonatal intensive care units [3] to the terminal stages of individuals in palliative care [4]. These interventions are often highlighted for their lack of adverse effects and their ability to reduce symptoms associated with illness and recovery, particularly those necessitating prolonged hospitalization [5]. Prolonged treatments often lead to stress, anxiety, depression, and other negative psychological effects. Music therapy within hospital settings emerges as a highly efficacious tool for mitigating these effects [6].

Music therapy is often used alongside cancer treatment. For example, studies by [5] and [7] explore the application of music therapy for cancer patients in hospitals. Another frequent application is in the context of haemodialysis treatments, which are chronic and typically involve regular hospital visits by patients [8,9]. Additionally, applications extend to cardiology, among other medical fields [10].

The concept of live music in hospitals requires clarification, as it can be interpreted in different ways. According to [11] live music in this context refers to the intentional delivery of music through the direct intervention of a music therapist, who may either perform themselves or engage another musician. In all cases, the music is delivered live in real time with therapeutic intent by a trained professional. Reference [12] describes this as person-centered music performed by a trained professional. This practice is commonly referred to as active music therapy [11]. This approach is also known as “live bedside music,” and several studies underscore its effectiveness [13,14]. This contrasts with passive music therapy, where patients listen to recorded music without direct interaction [15]. Examples of passive music therapy are included in numerous studies, such as those by [16,17,18]. Ref. [19] further distinguished this passive listening experience as music medicine, contrasting it with music therapy, which necessitates active human participation to maximize therapeutic outcomes.

Recorded music, enabled by technological advances, has become ubiquitous, allowing individuals to access music at any time. However, live music retains distinctive qualities that render it irreplaceable [20]. The physical presence of musicians conveys a depth of emotion that recordings often fail to capture. Moreover, the immediacy of instruments—their natural timbre and vibrations—resonates with listeners in a way that recorded sound cannot fully reproduce [21]. Empirical studies, such as that by [22], further highlight the unique physiological responses elicited by live music, underscoring its added impact on the human body.

Many music therapists highlight the value of active, live-based interventions due to their enhanced effectiveness [23]. Recent studies indicate that the use of live music in healthcare settings is becoming increasingly widespread [24]. According to Olmo Barros [25], when patients experience live music, they engage with it in a multisensory way: they not only hear the sounds but also perceive the vibrations produced by the voice and instruments, and visually follow the therapist’s movements during the performance. In this sense, patients do not merely listen to the music; they also witness its creation in real time, which may contribute to a deeper and more immersive therapeutic experience.

Other systematic reviews of the academic literature on music therapy in various healthcare settings and contexts have been conducted. For example, ref. [26] evaluated the use of music to enhance postoperative recovery following surgical procedures. Additionally, ref. [27] examined musical interventions for preoperative anxiety in surgical patients. Moreover, ref. [28] conducted a systematic review to assess the effect of music on anxiety and pain during surgical operations between 1980 and 2016. Another influential scoping review is the one by [1]. This seminal study examines the role of the arts in enhancing health and well-being within the hospital context.

This scoping review differs from previous reviews by focusing specifically on live music interventions. Earlier reviews have typically examined broader music-based interventions, including both active and passive approaches, in specific contexts such as postoperative recovery, preoperative anxiety, and surgery. In contrast, our review concentrates on live music interventions in order to better understand the contexts in which they have been used and the value they may bring to healthcare settings.

The aim of this study is to identify and synthesize the applications of live music in healthcare settings and to examine the reported benefits associated with these interventions. In particular, the review explores the clinical contexts in which live music has been implemented, the patient groups involved, the outcomes reported, and the countries in which these interventions have been studied. It also considers the value of live music as a practice that may contribute to the humanization of hospital care. This scoping review addresses the following research question: How has live music therapy been applied in healthcare settings, and what outcomes have been reported in relation to patients’ well-being and their clinical context?

## 2. Materials and Methods

### 2.1. Scoping Review

A scoping review is recommended when the aim is to identify research gaps, map the scope of the literature, and clarify concepts within a specific field [29]. Scoping reviews are particularly valuable when evidence on a relatively novel topic is still emerging [30]. This review follows the framework proposed by Arksey and O’Malley [31], who suggest refraining from establishing predefined limits at the outset of the investigation to identify the most relevant studies. The process is iterative, involving a retrospective examination of collected information to refine the obtained data. The scope review should be inclusive and informative, providing a broad overview of the research field.

A scoping review of studies examining live music in healthcare settings as an adjunct to clinical treatment was conducted to obtain a comprehensive understanding of the current evidence base. The protocol for this review has been registered on the Open Science Framework (OSF) under the title “Live Music in Hospital Settings and Reported Therapeutic Values: A Scoping Review Protocol”. The corresponding checklist is provided as Supplementary Materials to this article.

### 2.2. Studies Included for the Review

The search was conducted based on two conceptual blocks: “live music” and “healthcare settings.” Prior to this, several preliminary searches were performed to identify the most relevant terms and ensure accurate coverage of both concepts. The search strategy has been based on the following terms:

Keywords: For the live music concept (live music, live music therapy, active music therapy, interactive music therapy, bedside music, live bedside music, participatory music therapy, music live, live performance). For the healthcare setting concept (hospital, infirmary, healthcare, health care, clinic, ICU, NICU, ward, palliative care, oncology, rehab*, patient, haemodialysis, preterm infants, intensive care, cancer, newborn, geriatric, emergency, paediatric, psychiatri*).

Electronic databases: Web of science, Scopus and Pubmed.

Scope of the research: Articles from peer reviewed academic journals.

Timeframe: The search covered articles published from 2006 to 2026.

Language: English and Spanish.

Search strategies were adapted to the characteristics and indexing systems of each database using combinations of keywords and Boolean operators. Core search terms related to “live music” and healthcare contexts were combined primarily using the operators AND and OR to broaden or refine the retrieval process. Synonymous or closely related terms were linked with OR, while conceptual categories (e.g., live music and healthcare settings) were combined with AND. When appropriate, quotation marks and truncation symbols were also employed to improve search precision and capture variations in relevant terms across databases.

Limiting the review to the last 20 years allowed the inclusion of studies most relevant to current healthcare contexts while maintaining a manageable and methodologically feasible scope for the review.

### 2.3. Eligibility Criteria

Predefined inclusion and exclusion criteria were established to ensure consistency and transparency during the study selection process. These criteria were designed to identify studies specifically addressing live music interventions within healthcare settings while excluding publications not aligned with the objectives and scope of the review. The eligibility criteria applied during title, abstract, and full-text screening are detailed in Table 1.

### 2.4. Study Selection

**Figure 1:**
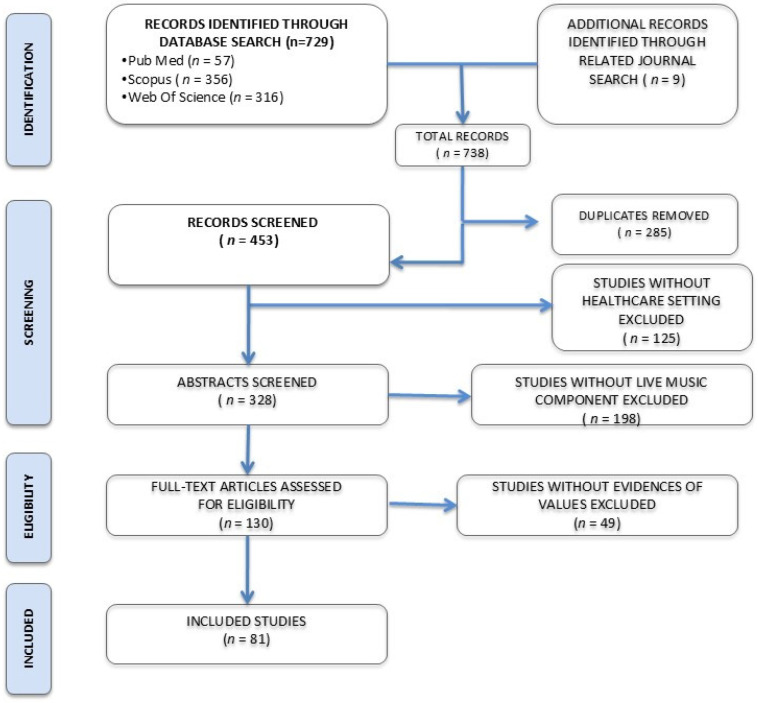
PRISMA-ScR flow diagram.

The initial search identified 729 records. In parallel, an additional manual search of related journals was conducted, identifying nine additional records. After removing duplicates and translated versions, titles and abstracts were screened for relevance, excluding studies not conducted in healthcare settings and those lacking a live music component. When eligibility remained unclear, full texts were reviewed and discussed among the researchers to ensure correct application of the inclusion and exclusion criteria. Most uncertainties concerned the nature of the musical intervention and the existence of reported evidence-based benefits. Following this process, 81 studies met the inclusion criteria. The study selection process is illustrated in the PRISMA-ScR flow diagram (Figure 1).

## 3. Results

### 3.1. Countries Represented in the Included Studies

A significant diversity of countries conducting studies involving live music is observed, with detailed information provided in Table 2.

### 3.2. Clinical Contexts and Healthcare Settings

Healthcare settings were grouped into five broad categories to provide a clearer and more interpretable overview of the contexts in which live music interventions have been implemented. This grouping was based on similarities in clinical purpose, patient population and care environment, rather than on the specific name of each hospital unit. For example, oncology and palliative care were grouped together because both frequently emphasize comfort, emotional support and quality of life, while neonatal, paediatric and maternity settings were combined due to their shared family-centred and developmental focus. This classification allowed the review to reduce excessive fragmentation across individual units while preserving the main differences between healthcare contexts. Specifically, the following Table 3 displays the studies grouped by the setting in which they were applied.

To facilitate interpretation and reduce excessive heterogeneity, individual study designs were grouped into four broader methodological categories based on similarities in research approach and evidence generation. Experimental quantitative studies included interventions using structured quantitative methods to evaluate outcomes, such as randomized or quasi-experimental designs. Observational quantitative studies comprised non-interventional designs using quantitative data collection, including surveys, cross-sectional studies, and observational analyses. Qualitative studies explored experiences, perceptions, meanings, and contextual aspects of live music interventions through interviews, observations, or thematic analyses. Mixed-methods studies combined quantitative and qualitative approaches to provide a more comprehensive understanding of interventions and their outcomes. This classification provides an overview of the predominant types of evidence identified in the literature and highlights the largely exploratory and descriptive nature of research on live music interventions in healthcare settings. Table 4 summarizes the methodological design of the studies included in the review. Appendix B presents each article individually, outlining the methodology and key characteristics of each study.

## 4. Discussion

In Section 3, we first group live music interventions in healthcare settings according to the clinical populations to which they are applied, providing an overview of which patients currently benefit from them. Furthermore, we classify these interventions based on the benefits obtained, regardless of the hospital setting, to determine the therapeutic value that can be attributed to live music. Considering the information from this dual perspective allows us to identify new gaps and potential applications of live music in healthcare settings that have not yet been explored.

### 4.1. Therapeutic Values Identified Across the Included Studies

Following an in-depth analysis of the benefits derived from live music across all contexts, the results were synthesized and grouped according to the nature of the reported positive values. This classification clearly highlights the multidimensional character of the benefits obtained. The synthesized values are presented and discussed below.

#### 4.1.1. Psychological Values

The evidence shows that live music-based interventions consistently produce psychological benefits across diverse clinical contexts. Studies report reductions in anxiety and perceived stress, as well as increases in relaxation understood as a psychological state. Live music has also been shown to support coping processes, helping patients to manage emotionally demanding situations such as hospitalization, treatment and recovery. Patients reported improved mood regulation and reduced psychological distress following live music exposure. Taking together, the literature indicates that live music can act as a meaningful psychological support during periods of clinical vulnerability.

#### 4.1.2. Well-Being Values

Across the reviewed literature, live music-based interventions are consistently associated with improvements in personal well-being during illness and treatment. Reported benefits include regulation of emotions, increased relaxation, pain reduction, improved mood and confidence, enhanced quality of life, and altered perception of time. These findings indicate that live music can meaningfully support the lived experience of patients by improving their perceived well-being during clinical care.

#### 4.1.3. Social and Emotional Benefits

Across the reviewed studies, live music-based interventions are consistently associated with social and emotional benefits for patients, families and staff. These include enhanced emotional expression, feelings of comfort and emotional support, reduced loneliness, and greater emotional connection with others. Reported outcomes also include improvement in social skills and self-esteem, assistance in verbalizing emotional states, and prevention of isolation. Live Music further promotes social engagement and interaction, helping to create shared experiences and strengthen relationships within care settings. Together, these findings indicate that live music can play an important role in supporting the emotional climate and social connectedness of people in clinical environments.

#### 4.1.4. Physical and Physiological Outcomes

Live Music-based interventions demonstrate measurable physical and physiological effects across clinical contexts. Reported outcomes include reinforcement of balance, coordination of muscle groups, motor skills, agility and endurance. Live Music can activate or sedate the body and has been shown to regulate respiratory and cardiac frequency, oxygen saturation and blood pressure. Evidence also indicates improvements in vital-sign stability, oxygenation, ventilator agitation and sedation needs, nausea and muscle tension, fatigue and overall autonomic regulation.

#### 4.1.5. Cognitive and Neurological Values

Live Music-based interventions demonstrate clear cognitive and neurological effects across clinical populations. Music can stimulate multiple brain areas simultaneously, including regions involved in memory, movement and attention, and it can modulate the speed and organisation of brain waves. Evidence shows activation and modulation of brain activity in people with disorders of consciousness (including EEG/aEEG/ERP responses), support for early neurological regulation and neurodevelopment in preterm infants and infants with perinatal brain injury, and improvements in core cognitive domains such as attention, memory and executive functioning during rehabilitation after traumatic brain injury. In people with Alzheimer’s disease, music has also been associated with enhanced goal-directed and attentional behaviour, indicating preserved or supported cognitive processing in the presence of music.

#### 4.1.6. Hospital Environment Values

The reviewed studies suggest that live music-based interventions can positively influence the hospital environment by creating a warmer, calmer, and more welcoming atmosphere for patients, families, and healthcare professionals. Live music has been reported to soften the clinical atmosphere, reduce perceived noise, and promote a calmer and more welcoming setting. These environmental effects extend beyond the individual patient, shaping the overall tone of care spaces and supporting a more humane and comforting experience within healthcare contexts.

We grouped the content of all studies and identified, through the screening process, the values reported in each study. Many studies exhibited several of these identified and classified values; therefore, the total count exceeds the sample size. Table 5 summarizes the values identified with the sample studies. Appendix A presents each article individually, specifying the values observed in each case.

### 4.2. Live Music vs. Recorded Music

An important theme emerging from the reviewed literature concerns the distinctive characteristics of live music compared with recorded music interventions in healthcare settings. While recorded music interventions are often designed primarily to achieve relaxation or distraction, live music appears to introduce additional relational, adaptive, and contextual dimensions. Several studies described how musicians adjusted tempo, repertoire, intensity, or interaction style in real time according to patients’ emotional responses, clinical conditions, or environmental dynamics. This adaptability was particularly relevant in neonatal intensive care, palliative care, and oncology settings, where emotional sensitivity and individualized care are central. Across multiple contexts, live music was also associated with broader processes of care humanization, including the creation of calmer atmospheres, enhanced interpersonal connection, emotional expression, and increased perceptions of comfort and dignity. These findings suggest that the potential value of live music in healthcare may extend beyond symptom-oriented outcomes toward experiential and relational dimensions of care that are less easily replicated through standardized recorded music interventions.

## 5. Conclusions

The use of live music as an aid in clinical treatments has increased in recent years and is employed by professionals across various pathologies as previously stated in the introduction and corroborated by our study. The findings of this review indicate a growing interest in the use of live music in healthcare settings. We also observe that the pathologies and hospital units examined in these studies are highly diverse.

This review suggests that live music interventions may contribute to healthcare across multiple interconnected dimensions extending beyond conventional symptom management. The identified psychological, physiological, cognitive, social, environmental, and well-being-related values reveal a multidimensional framework in which live music simultaneously supports patients’ emotional state, interpersonal relationships, and care environments. These effects may contribute to more humane and supportive healthcare experiences, potentially improving patient engagement and comfort, particularly in vulnerable populations such as oncology, palliative care, paediatrics, and geriatrics. The relational and environmental benefits reported across the literature may also positively influence families, caregivers, and healthcare staff, contributing to a more supportive atmosphere of care.

This scoping review encompasses the diversity of recent studies considered and highlights the need for continued research in this compelling field. The evidence base is still heterogeneous. Study designs, intervention types and outcome measures vary widely, and the mechanisms linking music to these benefits require further clarification. Rigorous randomised and longitudinal studies, alongside qualitative research exploring patient and staff perspectives, are needed to refine theoretical understanding and support effective clinical implementation. Future work should also consider how music can be ethically, equitably and sustainably integrated into routine care pathways, including who delivers it, how it is resourced and how outcomes are evaluated.

The findings of this review suggest that live music interventions may contribute to healthcare not only through psychological and emotional support, but also through broader processes of environmental improvement, interpersonal connection, and care humanization. Across diverse clinical settings, live music was associated with experiential and relational dimensions that align with growing patient-centred and holistic healthcare approaches, complementing conventional biomedical treatment through a non-pharmacological, low-risk, and adaptable intervention.

An important contribution of this review is the synthesis of the reported outcomes into six broad value dimensions—psychological and emotional, physical and physiological, cognitive and neurological, social and relational, environmental, and overall well-being-related values—highlighting the multidimensional nature of live music in healthcare environments. The reviewed literature also suggests that live music interventions can be adapted flexibly to different institutional contexts, patient populations, and clinical objectives. In settings such as neonatal and intensive care, interventions tended to emphasize individualized and carefully adapted musical interaction, whereas oncology, palliative care, and long-term care contexts more frequently highlighted emotional support, communication, dignity, and social connection.

These findings may help inform future hospital-based arts programs, interdisciplinary collaborations, and healthcare policies seeking to integrate more humanized and patient-centred care strategies in feasible and sustainable ways. Successful implementation appears to depend on collaboration among healthcare professionals, musicians, music therapists, hospital administrators, and community arts organizations. At the same time, the growing integration of live music into healthcare may also create new professional opportunities for musicians within clinical and community health contexts.

Despite the growing body of literature, the evidence base remains methodologically heterogeneous and predominantly exploratory. Future research should therefore strengthen methodological rigor, clarify conceptual definitions of live music interventions, and further investigate the distinctive mechanisms and contextual contributions of live compared with recorded music across healthcare settings.

### Limitations

Several limitations should be considered when interpreting the findings of this review. Although the search strategy included multiple databases, publications in English and Spanish, and a 20-year time frame, relevant studies published in other languages or indexed in additional sources may not have been captured. Beyond these procedural limitations, the evidence base itself presented substantial methodological and conceptual heterogeneity. The included studies varied considerably regarding intervention characteristics, healthcare settings, participant populations, performer roles, duration and frequency of interventions, and reported outcomes, limiting direct comparability across studies. In addition, the concept of “live music” was operationalized inconsistently across the literature, with differences in therapeutic intention, degree of interaction, and implementation format complicating synthesis and interpretation.

The reviewed evidence was also predominantly exploratory and descriptive, with many studies relying on qualitative approaches, observational designs, small sample sizes, or self-reported outcomes. Considerable variability in evaluation methods and outcome measures further limited the identification of consistent patterns across studies. Moreover, no formal methodological quality assessment was conducted, consistent with the exploratory objectives of scoping review methodology, meaning that studies of differing methodological rigor contributed equally to the synthesis. Finally, the predominance of positive findings in the literature may partially reflect publication or reporting biases within the field of arts and health. Consequently, the findings should be interpreted as a descriptive mapping of the existing evidence base rather than as definitive evidence of effectiveness.

## Figures and Tables

**Table 1:** Inclusion and exclusion criteria for the selection of articles for the study.

Element	Inclusion Criteria	Exclusion Criteria
Population	Patients of any age or clinical condition receiving or exposed to live music interventions in healthcare settings. Family members, caregivers, visitors, and healthcare professionals were also eligible when included as participants.	Populations not associated with healthcare settings.
Concept	Live music interventions involving real-time musical performance delivered in the presence of participants.	Recorded music interventions, music delivered exclusively through technological devices, or studies lacking a live music component.
Context	Any healthcare setting, including acute care hospitals, rehabilitation centres, hospices, long-term care facilities, neonatal units, outpatient services, and mental health facilities.	Community, educational, occupational, recreational, or other non-healthcare settings.
Evidence Sources	Empirical peer-reviewed articles using qualitative, quantitative, or mixed-methods approaches.	Reviews, editorials, commentaries, protocols, conference abstracts, and other publications.
Language	English and Spanish	Other languages.
Publication Period	Published during the last 20 years.	Published outside the specified period.

**Table 2:** Articles per country.

Number of Studies per Country	Countries
25	USA
8	Spain
6	Australia
5	Italy
4	Germany
3	Brazil, China, Netherlands, South Africa,
2	Canada, Ireland, Sweden, UK
1	Austria, Colombia, Denmark, Egypt, Finland, Switzerland, Hungary, Iran, Norway, Thailand

**Table 3:** Healthcare settings.

Healthcare Setting	Number	Studies
General hospital care	24	[10,14,32,33,34,35,36,37,38,39,40,41,42,43,44,45,46,47,48,49,50,51,52,53]
Critical and specialized care	27	[54,55,56,57,58,59,60,61,62,63,64,65,66,67,68,69,70,71,72,73,74,75,76,77,78,79,80]
Oncology and palliative care	17	[6,7,81,82,83,84,85,86,87,88,89,90,91,92,93,94,95]
Pediatric care	6	[96,97,98,99,100,101]
Rehabilitation and mental health care	7	[102,103,104,105,106,107,108]

**Table 4:** Study design types.

Study Design Types	Number	Studies
Experimental quantitative	55	[6,10,32,34,35,36,38,39,40,41,44,45,46,48,49,52,53,54,55,56,57,58,60,61,63,64,65,67,70,72,73,75,77,78,80,82,83,84,86,87,88,89,90,91,92,93,94,96,99,100,101,102,103,105,106]
Observational quantitative	11	[33,50,51,62,68,69,71,74,76,79,104]
Qualitative	8	[43,66,81,95,97,98,107,108]
Mixed methods	7	[7,14,37,42,47,59,85]

**Table 5:** Values and Sample studies.

Values	Number Studies	Sample Studies
Psychological outcomes assessed	48	[6,7,10,14,33,34,35,37,39,40,41,43,44,45,46,47,48,49,50,51,52,53,54,57,58,59,61,67,75,78,79,80,81,82,85,87,88,89,90,92,93,94,98,99,100,102,105,106]
Personal wellbeing	43	[10,14,33,34,36,37,38,40,41,42,44,48,54,55,56,59,62,63,64,66,67,72,73,77,80,81,82,83,84,85,86,87,88,89,92,93,94,101,103,104,106,107,108]
Social and emotional	34	[6,7,10,32,33,37,39,40,41,43,44,47,49,50,57,59,60,64,66,68,79,81,84,85,88,89,91,92,95,96,98,103,106,107,108]
Physical and physiological	27	[6,7,14,35,40,46,49,54,55,58,60,61,63,65,67,68,69,74,75,76,82,86,90,94,96,99,101]
Cognitive/Neurological	13	[6,39,41,56,59,60,65,70,71,72,103,104,107]
Hospital environment	24	[33,35,37,38,42,43,50,51,54,57,62,63,64,67,74,77,79,80,81,86,93,95,97,107]

## Data Availability

No new data were created or analyzed in this study.

## Supplementary Materials

The following supporting information can be downloaded at: https://www.mdpi.com/article/10.3390/healthcare14121805/s1, PRISMA 2020 Checklist.

## Appendix A. List of Included Studies with Own Values

**ID**	**Author (Year)**	**Country**	**Healthcare Condition/Setting**	**Psychological Outcomes**	**Personal Well Being**	**Social and Emotional**	**Physical and Physiological**	**Cognitive/Neurological**	**Hospital Environment**
[6]	Ramírez et al. (2018)	Spain	Palliative oncology	X		X	X	X	
[7]	Peng et al. (2019)	USA	Palliative unit	X		X	X		
[10]	Selle & Silverman (2017)	USA	Cardiovascular unit	X	X				
[14]	Fallek et al. (2020)	USA	Urban hospital	X	X		X		
[32]	Holmes et al. (2006)	U K	Dementia care setting			X			
[33]	Moss et al. (2007)	Ireland	University teaching hospital	X	X	X			X
[34]	Madson & Silverman (2010)	USA	Transplant unit	X	X				
[35]	Walworth (2010)	USA	Diagnostic MRI	X			X		X
[36]	Bradt (2010)	USA	Hospital postoperative care setting		X				
[37]	Canga et al. (2012)	USA	Chemotherapy	X	X	X			X
[38]	Mogos et al. (2013)	USA	General hospital units		X				X
[39]	Cox et al. (2014)	Australia	Residential aged-care facility	X		X		X	
[40]	Schneider et al. (2015)	USA	Mixed units	X	X	X	X		
[41]	Hogan & Silverman (2015)	USA	Transplant unit	X	X	X		X	
[42]	Harrop-Allin et al. (2017)	South Africa	Medical centre		X				X
[43]	Selle & Silverman (2018)	USA	Cardiovascular unit	X		X			X
[44]	Alcântara-Silva et al. (2018)	Brasil	Radiotherapy	X	X	X			
[45]	Van der Heijden et al. (2018)	South Africa	Burn wound care	X					
[46]	Teckenberg-Jansson et al. (2019)	Finland	Pregnant women unit	X			X		
[47]	Corey et al. (2019)	USA	Obstetric inpatient units	X		X			
[48]	Reimnitz & Silverman (2020)	USA	Blood and marrow transplant unit	X	X				
[49]	Alvarez et al. (2021)	Spain	NICU	X		X	X		
[50]	Reidy & MacDonald (2021)	USA	Acute hospital during COVID-19	X		X			X
[51]	Collins et al. (2022)	Ireland	Pain clinic	X					X
[52]	Xie et al. (2022)	China	Hematopoietic stem-cell transplantation	X					
[53]	Feng et al. (2024)	China	residential dementia care setting	X					
[54]	Walworth et al. (2008)	USA	Neurosurgical hospital unit	X	X		X		X
[55]	Sand-Jecklin & Emerson (2010)	USA	Mixed medical/surgical patients		X		X		
[56]	Bechert Caminha et al. (2011)	Brasil	Hemodialysis		X			X	
[57]	Malloch et al. (2012)	Australia	NICU/infant mental health context	X		X			X
[58]	Binson et al. (2013)	Thailand	Hospital haemodialysis units	X			X		
[59]	Homel et al. (2017)	USA	Postoperative spine surgery unit	X	X	X		X	
[60]	Ranger et al. (2018)	Germany & Switzerland	Neonatal ICU context			X	X	X	
[61]	Luis et al. (2019)	Egypt	Cardiothoracic surgery	X			X		
[62]	Morales-Betancourt et al. (2020)	Spain	Neonatal intensive care		X				X
[63]	Gelatti et al. (2020)	Italy	Ambulatory surgery		X		X		X
[64]	Van Dokkum et al. (2020)	Netherlands	NICU (tertiary centre)		X	X			X
[65]	Span et al. (2021)	Netherlands	Neonatal intensive care				X	X	
[66]	Chifa et al. (2021)	Brasil	Neonatal Intensive Care Units (NICUs)		X	X			
[67]	Menke et al. (2021)	Germany	NICU	X	X		X		X
[68]	Meder et al. (2021)	Hungary	NICU			X	X		
[69]	Kobus et al. (2021)	Germany	NICU				X		
[70]	Giordano et al. (2021)	Austria	NICU					X	
[71]	Bos et al. (2021)	Netherlands	NICU					X	
[72]	Namjoo et al. (2021)	Iran	NICU		X			X	
[73]	Soliva et al. (2022)	Spain	Hemodialysis		X				
[74]	Kobus et al. (2022)	Germany	NICU				X		X
[75]	Golino et al. (2023)	USA	Intensive care unit	X			X		
[76]	Brown et al. (2023)	Canada	NICU				X		
[77]	Rossetti et al. (2023)	USA	SICU/PICU environment		X				X
[78]	Soliva et al. (2024)	Spain	Hemodialysis units	X					
[79]	Burrai et al. (2025)	Italy	Haemodialysis unit	X		X			X
[80]	Hernandez et al. (2025)	Colombia	Emergency Department (ED) waiting areas	X	X				X
[81]	O’Callaghan & Magill (2009)	Australia	Two cancer centers	X	X	X			X
[82]	Chaput-McGovern & Silverman (2012)	USA	Surgical oncology unit	X	X		X		
[83]	Wormit et al. (2012)	Germany	Oncology service		X				
[84]	Gutgsell et al. (2013)	USA	Advanced illness (hospice/palliative)		X	X			
[85]	Gatto et al. (2017)	Canada	Hematology–oncology inpatient uni	X	X	X			
[86]	Burrai et al. (2014)	Italy	Cancer hospital ward		X		X		X
[87]	Warth et al. (2015)	Germany	Palliative care unit	X	X				
[88]	Augé et al. (2015)	Spain	Psycho-oncology	X	X	X			
[89]	Palmer et al. (2015)	USA	Breast cancer surgical setting	X	X	X			
[90]	Warth et al. (2016)	Germany	Palliative care	X			X		
[91]	Cadwalader et al. (2016)	USA	Hospice care facility			X			
[92]	Warth et al. (2018)	Germany	Palliative care	X	X	X			
[93]	Toccafondi et al. (2018)	Italy	Oncology wards	X	X				X
[94]	Bro et al. (2019)	Dennmark	Oncology day-unit	X	X		X		
[95]	Apps & Sunderland (2021)	Australia	Hospital oncology units			X			X
[96]	Del Olmo et al. (2010)	Spain	Pediatric Intensive Care Unit (PICU)			X	X		
[97]	Preti and Welch (2012)	Italy	Paediatric hospital						X
[98]	Bower et al. (2014)	Australia	Pediatric neuro-rehabilitation	X		X			
[99]	Uggla et al. (2016)	Sweden	Pediatric HSCT unit	X			X		
[100]	Scheufler et al. (2021)	USA	Pediatric pain unit	X					
[101]	Mata Ferro et al. (2023)	Spain	Pediatric intensive care unit		X		X		
[102]	Morgan et al. (2011)	Australia	Psychiatric unit	X					
[103]	O’Kelly et al. (2013)	United Kingdom	Neurorehabilitation		X	X		X	
[104]	Zhang et al. (2015)	China	Neuro-rehabilitation setting		X			X	
[105]	Wood et al. (2021)	USA	Rehabilitation unit	X					
[106]	Madsø et al. (2022)	Norway	Long-term care	X	X	X			
[107]	Wijk et al. (2022)	Sweden	Long-term residential care		X	X		X	X
[108]	Stuart-Röhm et al. (2023)	South Africa	long-term dementia care facilities		X	X			

## Appendix B. List of Included Studies with Study Design Characteristics

**ID**	**Author (Year)**	**Study Design**	**Sample Size**	**Intervention Dosage**	**Measurement Tools**
[6]	Ramírez et al. (2018)	Randomized controlled trial	40 patients	One music therapy session including passive listening (C1), active listening (C2), and relaxation (R) phases	Electroencephalography (EEG); arousal-valence emotional model; pre/post questionnaires assessing tiredness, anxiety, breathing difficulties, and well-being
[7]	Peng et al. (2019)	Mixed-methods study (quantitative pre-post assessment and qualitative narratives)	46 patients	One live music intervention	Edmonton Symptom Assessment Scale (ESAS);
[10]	Selle & Silverman (2017)	Experimental randomized pretest-posttest wait-list control study	36 patients	Single-session Patient-Preferred Live Music	Quick Mood Scale; 10-point Likert pain scale
[14]	Fallek et al. (2020)	Mixed-methods pre-post study	150 patients	One bedside music therapy session	Pre-post assessments of anxiety, pain, pulse, and respiratory rate
[32]	Holmes et al. (2006)	Randomized placebo-controlled trial with blinded observer rating	32 participants	30-min sessions of live music,	Dementia Care Mapping
[33]	Moss et al. (2007)	observational evaluation study	Not available	Repeated live orchestral performances within the hospital	Perception-based evaluation methods
[34]	Madson & Silverman (2010)	Pre-posttest pilot study	58 patients	individual music therapy session lasting 15–35 min consisting of live patient-preferred music	Self-reported 10-point Likert-type scales for anxiety, relaxation, pain, and nausea
[35]	Walworth (2010)	Comparative controlled study	88 patients	Live music therapy delivered throughout MRI scan procedure	Patient perception of MRI procedure; number of repeated scans due to movement
[36]	Bradt (2010)	Within-subjects counterbalanced crossover design with randomized treatment sequences	32 pediatric patients	Two music entrainment sessions	Pre- and post-condition assessments of pain perception and emotional state
[37]	Canga et al. (2012)	Pilot mixed-methods study (quantitative survey + qualitative phenomenological analysis)	52 surveys: 27 patients, 10 caregivers, 15 staff members	Weekly 30-min EMT sessions over a 4-month period	Custom EMT survey evaluating environmental impact, perceived volume, music preferences and open-ended responses; qualitative interpretative phenomenological analysis (IPA)
[38]	Mogos et al. (2013)	Quasi-experimental two-group comparison study	100 patients	Bedside live therapeutic music session	Brief questionnaire assessing affect and perceptions of care; structural equation modelling
[39]	Cox et al. (2014)	Exploratory one-group repeated-measures study	7 participants	Three individual live violin sessions per participant	Video analysis; investigator-modified Cohen-Mansfield Agitation Inventory focused on 16 positive behaviours; blinded assessment
[40]	Schneider et al. (2015)	two-treatment crossover randomized clinical trial	92 patients	One session of 30–40 min of prescriptive live therapeutic harp music	Linear analogue scales measuring quality of life, well-being, relaxation, fatigue, anxiety, sadness, and pain
[41]	Hogan & Silverman (2015)	Randomized pretest-posttest wait-list control pilot study	25 patients	One 30-min patient-preferred live music	Positive affect measures; negative affect measures; pain assessment; pretest-posttest ANCOVA analyses
[42]	Harrop-Allin et al. (2017)	Mixed-method programme evaluation	Not available	Ongoing live music performances delivered through a student placement programme	Questionnaires, nurse focus groups, student focus groups, and student reflective essays
[43]	Selle & Silverman (2018)	Exploratory qualitative interpretivist study using thematic analysi	10 patients	Two Patient-Preferred Live Music	ndividual semi-structured interviews
[44]	Alcântara-Silva et al. (2018)	Randomized controlled trial	164 randomized; 116 analyzed (63 control, 53 music therapy)	Individual 30–40 min music therapy sessions; average of 10 sessions per participant	Functional Assessment of Cancer Therapy–Fatigue (FACT-F v4); Functional Assessment of Cancer Therapy–General (FACT-G v4); Beck Depression Inventory (BDI)
[45]	Van der Heijden et al. (2018)	Randomized assessor-blinded controlled trial	135 patients	One live music therapy session immediately after the first or second wound care procedure	Observational Scale of Behavioral Distress-Revised (OSBD-r); COMFORT-Behavioral Scale (COMFORT-B); Wong-Baker FACES Scale; Faces Pain Scale-Revised (FPS-R); blinded video-based assessments
[46]	Teckenberg-Jansson et al. (2019)	Randomized controlled trial	102 women	Live music therapy for 30 min per day on three consecutive days	Twelve HRV measures (including SD2 and LF indices);
[47]	Corey et al. (2019)	Retrospective service evaluation with mixed quantitative and qualitative analysis	320 patients	One individualized bedside music therapy session	Post-intervention feedback questionnaires;
[48]	Reimnitz & Silverman (2020)	Randomized pilot study	35 patients	Single-session Patient-Preferred Live Music (PPLM) intervention	Lee Fatigue Scale; 10-point Likert-type pain scale
[49]	Alvarez et al. (2021)	Quasi-experimental repeated-measures study	44 infant-parent dyads	One live music therapy session	Heart rate (HR); oxygen saturation (O_2_-sat);
[50]	Reidy & MacDonald (2021)	Descriptive clinical report/practice paper	Not available	Not available	Not reported
[51]	Collins et al. (2022)	Multimethod survey study (cross-sectional comparative design)	200 adult patients	Exposure to live music in one waiting room	Self-administered questionnaire with rating scales and open-ended questions;
[52]	Xie et al. (2022)	Controlled quasi-experimental study with random assignment and repeated follow-up assessments	62 patients	Four-week live music therapy program	Hospital Anxiety and Depression Scale–Depression subscale (HADS-D
[53]	Feng et al. (2024)	Quasi-experimental study with control group	121 participants	12 music therapy sessions over 6 weeks	Anxiety and depression assessment scales
[54]	Walworth et al. (2008)	Randomized controlled trial	27 patients	One preoperative live music therapy session plus daily postoperative sessions until discharge	Visual Analog Scales (VAS) for anxiety, mood, pain,
[55]	Sand-Jecklin & Emerson (2010)	Exploratory study		Live music intervention during hospitalization	Pain, anxiety, and muscle tension assessments
[56]	Bechert Caminha et al. (2011)	Exploratory pre-post study	43 haemodialysis patients	Two live music improvisation sessions	Pre- and post-intervention assessment of subjective states of mind and perception
[57]	Malloch et al. (2012)	Controlled comparative study with hospitalized intervention group	39 infants	3 sessions/week	NAPI (Neurobehavioral Assessment of the Preterm Infant); ADBB (Alarm Distress Baby Scale)
[58]	Binson et al. (2013)	Comparative intervention study (live music vs music listening) with pre-post measurements	54 haemodialysis patients		Blood pressure measurements; pulse rate measurements; pain assessment; anxiety assessment
[59]	Homel et al. (2017)	Randomized controlled mixed-methods study	60 patients	Music therapy sessions delivered postoperatively	Visual Analog Scale (VAS); Hospital Anxiety and Depression Scale (HADS); Tampa Scale for Kinesiophobia
[60]	Ranger et al. (2018)	Two-centre randomized controlled crossover trial	21 preterm infants		Continuous physiological monitoring; HRV analysis (pNN50, SDNN); oxygen saturation monitoring
[61]	Luis et al. (2019)	Randomized controlled pilot study	12 patients	Two 20-min sessions of improvised, personalized live oud music	Anxiety scores; pain scores; vital signs (heart rate, respiratory rate); serum cortisol levels
[62]	Morales-Betancourt et al. (2020)	Prospective observational feasibility study	29 preterm infants	Live music delivered 3 times per week over one year	Newborn Infant Parasympathetic Evaluation (NIPE)
[63]	Gelatti et al. (2020)	Pilot quasi-experimental study	46 patients	One preoperative music session immediately before surgery	Self-report fear and stress questionnaire; heart rate and blood pressure measurements before and after intervention
[64]	Van Dokkum et al. (2020)	Single-centre feasibility study	18 preterm infants	Three live music therapy sessions per week,	COMFORT-Neo scale; parent questionnaires; nurse questionnaires; participation and dropout analysis
[65]	Span et al. (2021)	Prospective intervention study	17 very preterm infants	Six LPMT sessions;	Heart rate monitoring; respiratory rate monitoring; oxygen saturation monitoring;
[66]	Chifa et al. (2021)	Qualitative phenomenological study	12 interview participants + observational data from 3 NICUs		Participant observation, oral history interviews, field diaries, hermeneutic phenomenological analysis
[67]	Menke et al. (2021)	Randomized controlled longitudinal trial	65 parent–infant pair	Live-improvised music therapy twice weekly	parental stress questionnaires; regression and factor analyses
[68]	Meder et al. (2021)	Prospective single-centre observational cohort study	31 preterm infants	20 min live maternal singing + live guitar music during SSC	Near-infrared spectroscopy (NIRS), heart rate monitoring, oxygen saturation monitoring, cerebral fractional tissue oxygen extraction measurements
[69]	Kobus et al. (2021)	Prospective observational study nested within an ongoing randomized controlled trial	20 preterm infants	Live music therapy twice weekly	Heart rate monitoring, respiratory rate monitoring, pulse oximetry, behavioural assessment of wakefulness state
[70]	Giordano et al. (2021)	Randomized controlled trial	64 preterm infants	One 20-min live	Amplitude-integrated electroencephalography (aEEG) evaluating sleep cyclicity and neurophysiological maturation
[71]	Bos et al. (2021)	Pilot pre-post observational study with blinded assessment	12 infants	Approximately 15 min of individualized live music therapy	General Movement Optimality Score (GMOS) based on blinded video assessment of spontaneous movements
[72]	Namjoo et al. (2021)	Randomized clinical trial	90 preterm infants	20 min/day for 14 days	Physiological monitoring (heart rate and oxygen saturation) and infant sleep checklists
[73]	Soliva et al. (2022)	Prospective group-randomized intervention study	90 patients	Live classical music during hemodialysis sessions over 4 weeks	Kidney Disease Quality of Life Questionnaire (KDQOL-SF)
[74]	Kobus et al. (2022)	Prospective observational cohort study	40 preterm infants	Live music therapy twice weekly	Heart rate monitoring, respiratory rate monitoring, pulse oximetry, multivariable regression analysis
[75]	Golino et al. (2023)	Randomized controlled trial	118 patients	One 30-min individualized live music therapy session	Richmond Agitation-Sedation Scale (RASS); Critical Care Pain Observation Tool (CPOT); heart rate monitoring; respiratory rate monitoring; oxygen saturation measurement
[76]	Brown et al. (2023)	Pre–post observational study	106 neonates	Standard music therapy session (~15 min)	Distress assessment; heart rate monitoring; oxygen saturation monitoring
[77]	Rossetti et al. (2023)	Baseline randomized environmental study	233 participants (41 patients, 81 caregivers, 111 healthcare staff)	Comparison of randomized periods with no music versus live Environmental Music Therapy	Surveys assessing perception of noise and environmental experience among patients, caregivers, and staff
[78]	Soliva et al. (2024)	Randomized clinical trial	90 patients	Live classical music during haemodialysis sessions for 4 weeks	Anxiety scale; Depression scale
[79]	Burrai et al. (2025)	Observational evaluation study	122 participants (89 patients, 33 healthcare providers)	Live music for 30 min daily during six consecutive days	-point Likert scales assessing satisfaction and appropriateness;
[80]	Hernandez et al. (2025)	Pragmatic multicenter randomized clinical trial (3-arm)	430 participants total (256 patients, 174 caregivers)	Live EMT vs prerecorded music	State-Trait Anxiety Inventory (STAI-6); Visual Analog Stress Scale (VASS); Visual Analog Pain Scale (VAPS); Well-Being Numerical Rating Scales (WB-NRS)
[81]	O’Callaghan & Magill (2009)	Qualitative study using grounded theory methodology; two linked studies (open-ended questionnaire and interviews)	Study 1: 38 staff respondents; Study 2: 62 staff interviewees	Ongoing exposure to/witnessing patient-centered music therapy programs	Anonymous open-ended questionnaire; semi-structured interviews
[82]	Chaput-McGovern & Silverman (2012)	Pilot pretest-posttest follow-up study	27 oncology patients	One 20-min music therapy session consisting of patient-preferred live music; follow-up assessment conducted 30–45 min later	Five separate 10-point Likert-type scales measuring relaxation, pain, anxiety, nausea
[83]	Wormit et al. (2012)	Pre-post intervention study	20 cancer patients	20 individual music therapy session	Self-reported general therapy outcome measures; quality-of-life assessment; subjective pain ratings
[84]	Gutgsell et al. (2013)	Randomized controlled trial	200 palliative care inpatients	One music therapy session including live music and guided relaxation	Numeric Rating Scale (NRS); Face, Legs, Activity, Cry, Consolability Scale (FLACC); Functional Pain Scale (FPS)
[85]	Gatto et al. (2017)	Mixed-methods exploratory intervention study	7 adolescents	Four individual interactive music therapy sessions	Repeated psychosocial measures across seven time points; open-ended qualitative questions
[86]	Burrai et al. (2014)	Randomized controlled trial	52 cancer patients	One 30-min live saxophone performance	Physiological measurements (blood pressure, pulse, oxygen saturation, glycemia) and mood/pain assessments before and after intervention
[87]	Warth et al. (2015)	Randomized controlled trial	84 patients	Two sessions of live music–based relaxation exercise	Visual Analog Scales (VAS) for relaxation, well-being, and acute pain
[88]	Augé et al. (2015)	Pre-post intervention study	11 Patients	8 weekly group music therapy sessions	Hospital Anxiety and Depression Scale (HADS); Profile of Mood States (POMS-A); EORTC-QLQ-C30 Quality of Life Questionnaire
[89]	Palmer et al. (2015)	Randomized controlled trial (3 arms)	207 women	Preoperative music session and intraoperative music exposure during surgery	Anxiety scales; propofol consumption; recovery time; patient satisfaction measures
[90]	Warth et al. (2016)	Randomized controlled trial	84 patients	Two sessions of live music–based relaxation exercise	Vagally mediated heart rate variability (VM-HRV); blood volume pulse amplitude (BVP-A)
[91]	Cadwalader et al. (2016)	Single-blind pretest-posttest pilot study	77 participants	One 20-min live music therapy session	Overt Agitation Severity Scale (OASS)
[92]	Warth et al. (2018)	Pilot feasibility and pre-post intervention study	15 patients	Two consecutive sessions: (1) biographical interview and song selection; (2) live performance of the selected biographically meaningful song in lullaby style	Brief self-report measures assessing life closure
[93]	Toccafondi et al. (2018)	Controlled prospective study	242 cancer patients	One live music concert session	Distress Thermometer (DT); Hospital Anxiety and Depression Scale (HADS);
[94]	Bro et al. (2019)	Three-arm randomized controlled trial	143 patients	30-min patient-preferred live music	Spielberger State Anxiety Inventory; blood pressure; pulse rate; nausea and vomiting assessments; serum catecholamine measurements
[95]	Apps & Sunderland (2021)	Qualitative study	Not available	Live music performances within	Observations and semi-structured interviews analysed using inductive and theory-driven thematic analysis
[96]	Del Olmo et al. (2010)	Comparative repeated-measures study	100 interventions	Live music interaction sessions compared with non-music interaction periods	Heart rate monitoring; oxygen saturation monitoring; respiratory rate monitoring
[97]	Preti and Welch (2012)	Qualitative study using Grounded Theory methodology	20 healthcare professional	Regular exposure to live music	Semi-structured interviews
[98]	Bower et al. (2014)	Single-case study	1 paediatric patient	Music therapy sessions using live familiar songs	Video micro-analysis and Agitated Behaviour Scale (ABS)
[99]	Uggla et al. (2016)	Randomized clinical pilot study	24 pediatric HSCT patients	Music therapy sessions twice weekly	Routine hospital physiological monitoring
[100]	Scheufler et al. (2021)	Three-period, three-treatment crossover study with quasi-randomized intervention order	48 patients	Three music therapy interventions delivered	State-Trait Inventory for Cognitive and Somatic Anxiety for Children (state form); Visual Analog Scale (VAS) for relaxation
[101]	Mata Ferro et al. (2023)	Quasi-experimental pretest–posttest study	259 PICU patients	Single live music therapy session	Vital signs monitoring (heart rate, respiratory rate); discomfort and pain assessment scales
[102]	Morgan et al. (2011)	Quasi-randomized controlled study	60 participants	Music therapy adjunct to standard pharmacological treatment	Brief Psychiatric Rating Scale (BPRS); Calgary Interview Guide for Depression; Nurses’ Observation Scale for Inpatient Evaluation (NOSIE-30); Depression Anxiety Stress Scale (DASS21);
[103]	O’Kelly et al. (2013)	Controlled neurophysiological and behavioural experimental study	41 participants	Exposure to live preferred music, live improvisation entrained to respiration	Electroencephalography (EEG), heart rate variability, respiration monitoring, behavioural observation (including blink rate)
[104]	Zhang et al. (2015)	Feasibility and pilot evaluation study	26 participants	Six-week live music programme	Validated well-being measures for staff; repeated patient outcome assessments at five time points from baseline to follow-up
[105]	Wood et al. (2021)	Pilot feasibility study with pre-post assessments	20 patients	Two individualized 20-min MAR sessions	Visual Analog Scales (VAS) for pain, anxiety, and relaxation before and after each session; Generalized Anxiety Disorder Scale (GAD-7); Perceived Stress Scale (PSS-10); patient satisfaction questionnaire
[106]	Madsø et al. (2022)	Replicated single-case experimental design	11 dementia-caregiver dyads	Active music therapy intervention over 10 weeks;	Observable Well-being in Living with Dementia Scale (OWLS); Verbal and Nonverbal Sociable Interaction Scale–Care Receiver (VNVIS-CR)
[107]	Wijk et al. (2022)	Qualitative study using thematic analysis		Musical performances held across 11 nursing home	Semi-structured interviews with residents, relatives and caregivers
[108]	Stuart-Röhm et al. (2023)	Action research with iterative co-design cycles and qualitative evaluation	8 caregivers across 2 care homes	Four iterative cycles of PCCS training and implementation	Semi-structured group interviews
